# The psychometric properties of the of the Persian version of the Screen for adult anxiety related disorders (SCAARED) in patients with anxiety disorders

**DOI:** 10.1002/brb3.2647

**Published:** 2022-06-17

**Authors:** Abbas Ramezani Farani, Seyed Ali Khabaz, Maryam Nabavi, Roaya Didehvar, Samira Masuomian, Mitra Zahiriyan

**Affiliations:** ^1^ Mental Health Research Center, Tehran Institute of Psychiatry—School of Behavioral Sciences and Mental Health Iran University of Medical Sciences Tehran Iran

**Keywords:** anxiety disorders, psychometric properties, reliability, validity

## Abstract

**Background:**

The aim of this study was to investigate the psychometric properties of the Persian version of the Screen for adult anxiety related disorders (SCAARED) in Tehran.

**Method:**

The present study was a descriptive‐survey method and a cross‐sectional method. The present research population consists of patients referring to hospitals and psychiatric clinics in Tehran, as well as male and female students in Tehran. The sample of the present study included 300 participants (150 patients with a diagnosis of anxiety disorders and 150 non‐clinical samples), who were selected by random sampling method. Inclusion criteria included age 18 to 50, minimum diploma, lack of mental retardation, and lack of acute physical illnesses such as cancer or severe pain. The participants, after completing the demographic questionnaire and conducting a Structured Clinical Interview for DSM‐5 Disorders–Clinical Version (SCID‐5‐CV), completed the SCAARED and the Personal Wellbeing Index—Adults (PWI‐A). Finally, face and content validity and construct validity, test–retest reliability, Cronbach's alpha, and factor analysis were used.

**Results:**

The results of the present study confirmed the face validity and content of the present scale. A review of Cronbach's standardized alpha showed that SCAARED has a reliability of 0.966, and therefore, the Persian version of these questionnaires is a reliable tool. Also, the results showed a correlation between the two implementations of the questionnaire; in addition to the strong correlation at the level (*p *< .01) between the factors of the questionnaire and the factors with the total score, there was a strong correlation between the first and second implementation in four factors and the overall score. Therefore, it can be concluded that the SCAARED has good test–retest reliability. Also, there is a positive correlation between the factors and the overall score of the SCAARED with anxiety disorders based on Structured Clinical Interview for DSM‐5 Disorders (*p* < .01), which indicates the favorable convergent validity of the SCAARED questionnaire. There is a negative correlation between the factors and the overall score of the SCAARED with the PWI‐A at the level (*p* < .01), which indicates the favorable divergent validity of the SCAARED, and the results of exploratory factor analysis of the questionnaire were confirmed.

**Conclusion:**

The Persian version of the SCAARED is a tool with appropriate validity and reliability.

## Graphical Abstract

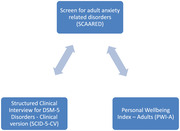

## 1 INTRODUCTION

Anxiety disorders are one of the most common psychiatric disorders (above 25%) among adults (Remes et al., 2016) that have negative effects on social functioning, mental and physical health, family and social relationships, and quality of life (Barlow, 2004; Kessler et al., 2007). Anxiety disorders also increase the risk of other disorders such as major depression and substance abuse (Khan et al., 2002; Leon et al., 1995).

In recent years, and through numerous studies, good tools have been proposed to evaluate, measure, and screen these disorders; nevertheless, in psychiatric and mental health settings, this class of disorders is less diagnosed and treated than it is (Combs & Markman, 2014; Kroenke et al., 2007). One reason for this is that the symptoms of these disorders overlap with other disorders or the symptoms of anxiety are ignored (Barnes et al., 2002; First, 1997), and therefore, the use of accurate and valid tools is necessary. Structured interviews such as the Anxiety Disorders Interview Program (Brown et al., 1994) and structured interviews for DSM‐IV (SCID‐IV) are good diagnostic tools used to assess anxiety disorders, but these tools require detailed and extensive training and a lot of time to implement (Antony & Rowa, 2005).

In addition, self‐report tools for measuring anxiety symptoms (Crocetti et al., 2009; Lowe & Reynolds, 2004; Monga et al., 2000) and measuring specific anxiety disorders such as social anxiety disorders (SAD) and generalized anxiety disorders (GAD) (Boyd et al., 2003; Crocetti et al., 2009; Hale et al., 2011; Plummer et al., 2016; Wren et al., 2007); but the Screen for adult anxiety related disorders (SCAARED) evaluates all DSM‐5 disorders and the symptoms of the disorders are based on DSM‐5. Hence, it can be used in research and clinical work. Therefore, due to the need for accurate assessment of anxiety disorders and the importance of psychological tools along with interviews in clinical and therapeutic work as well as in research work and on the other hand due to the gap in current tools for anxiety disorders that can address all anxiety disorders. Considering the DSM‐5, in this study, the aim is to investigate the psychometric properties of the SCAARED in patients with anxiety disorders in society of Iran.

## 2 METHODS

### 2.1 Populations

The present research population consists of patients referring to hospitals and psychiatric clinics (including Iran Psychiatric Hospital, Rasoul Akram Hospital, and Clinic of Behavioral Sciences and Mental Health [Tehran Psychiatric Institute]), as well as male and female students in Tehran. The sample of the present study included 300 participants (150 patients with a diagnosis of anxiety disorders and 150 non‐clinical samples) who were selected by random sampling method.

Also, the non‐clinical sample was randomly selected from among the students of Iran University of Medical Sciences. The colleagues of the project with at least a bachelor's education introduced the project and stated the purpose of the research and then invited people to participate in the project, and after reviewing the entry criteria, participants entered the research. Inclusion criteria in the present study are age range 18 to 50 years and minimum diploma education (due to the ability to complete the questionnaires) and written informed consent. After receiving the code of ethics from Iran University of Medical Sciences (IUMS) and the necessary coordination with the psychiatric centers, and after obtaining the cooperation and consent of individuals and receiving informed consent from the participants, and conducting a Structured Clinical Interview for DSM‐5 Disorders–Clinical Version (SCID‐5‐CV), the participants completed the demographic questionnaire, the SCAARED, and the Personal Wellbeing Index—Adults (PWI‐A).

### 2.2 Procedure

In this study, two steps were performed to translate and validate SCAARED. In the first step, the translation process and the concept of the questionnaire were performed and in the second, the validity and reliability of the questionnaire were investigated. To translate the SCAARED questionnaire, first two translators (translators 1 and 2) who were familiar with the field of psychology and questionnaire construction but had not yet seen the present questionnaire independently translated the questionnaire from English to Persian. Each translator provided a translation of the test items and a list of possible alternative translations. In a joint session with the presence of the main researchers of the project and translators, a single translation of the questionnaire was prepared. Then, two English language experts (translators 3 and 4) translated the Persian version of the questionnaire into English. In a joint meeting with the presence of the main researchers of the project and translators, the similarity of this version and the original version of the test was reviewed and the required corrections were made. After checking the face validity, the final version was prepared and used to collect information. The validity of the questionnaire was assessed through the methods of face and content, convergent and divergent, and construct validity and factor analysis. In order to check the face validity of the Persian version of the questionnaire, this test was given to five psychologists and psychiatrists who had good experiences in the field of anxiety disorders. After collecting information, the Lawshe method was used to determine the content validity. In this method, two indicators of content validity ratio (CVR) and content validity index (CVI) are used. In the Lawshe method, the content validity ratio index is calculated on a three‐point graph. Experts are asked to rate the importance and necessity of each items in the questionnaire (based on three modes: 1 = not necessary, 2 = useful but not necessary, and 3 = necessary). According to Lawshe method, if more than half of the experts determine that the existence of an item is necessary, that item has the minimum content validity (Lawshe, 1975). The more experts rated a particular item in terms of necessity, the higher the level of content validity of that item. In the Waltz and Bausell method (Waltz & Bausell, 1981), CVI was calculated. For this purpose, each item of this questionnaire was examined based on the three concepts of relevance, clarity, and simplicity on a four‐point chart and based on a 4‐part Likert scale. The minimum acceptable value for the CVI index is 0.79 (Munro, 2005).

Then, convergent validity was assessed by correlating the SCAARED with the SCID‐5‐CV diagnoses. To evaluate the reliability of SCAARED, the test–retest method and Cronbach's alpha were used. In the test–retest method, among the sample members, 30% of those who agreed to complete the test were re‐evaluated after 2 weeks.

### 2.3 Measurements

Demographic questionnaire: This researcher‐made questionnaire was used to obtain demographic information of patients, which includes items such as age, sex, marital status, employment status, educational status, and previous history and duration of psychiatric and psychological disorders (clinical and personality disorders) and received treatments (pharmacological and non‐pharmacological).

SCID‐5‐CV: This tool is a semi‐structured clinical and diagnostic interview developed by First et al. in 2015 to assess clinical disorders (First et al., 2016) including anxiety and mood disorders, psychosis, and substance abuse. Using this tool, the level of damage and severity of each disorder can be assessed. The validity and reliability of this tool have been examined and confirmed (Shabani et al., 2021).

SCAARED: A 44‐item scale that includes questions to assess the symptoms of anxiety disorders consistent with DSM‐5 disorders (agoraphobia disorder, GAD, SAD, panic disorder, separation anxiety disorder). The base range is from 0 (not true at all) to 2 (very true about). In a study by Angela et al. (Angulo et al., 2017), the results indicated that SCAARED had good internal consistency, and that SCAARED had good psychometric properties in support of its use in screening for anxiety disorders in adults (Angulo et al., 2017).

PWI‐A: The PWI‐A contains seven items of satisfaction, each related to an area of quality of life, including material level of life, health, personal relationships, safety, achievements in life, position, and social security in the future. Each of the scale questions is scored by the subject between 0 and 10 (Cummins & Lau, 2005). In Iran, the research findings showed that the scale has good reliability based on the complete alpha coefficient (0.89) and correlation coefficient and related to its retest (0.79). Also, the obtained correlation coefficients indicate the convergent validity of the scale with similar instruments (Nainian et al., 2014).

### 2.4 Statistical analysis

To analyze the research data, first the face and content validity was examined. Then, to determine that the factors of the present questionnaire are consistent with the factors expressed by the constructors, we used exploratory factor analysis and through Cronbach's alpha, internal validity was examined; through Pearson correlation, test–retest reliability, convergent and divergent validity, and correlation between factors were examined.

## 3 RESULTS

In the present study, the data obtained from 247 participants were analyzed in two groups of anxiety disorders with 107 participants (43.3%) and non‐clinical with 140 participants (56.7%) (53 samples were excluded from the final analysis due to incomplete tests or lack of cooperation). No cases of schizophrenia and other psychotic disorders were observed in the clinical group. In this group, 12 participants (11.2%) have obsessive‐compulsive disorder (OCD), 3 participants (2.8%) have bipolar disorder, 7 participants (6.5%) have substance abuse, 31 participants (29%) have several simultaneous disorders, 77 participants (72%) with GAD, 54 participants (50.5%) with panic disorder, 26 (24.3%) with separation anxiety disorder, 79 participants (73.8%) with social anxiety disorder (ASD), and 6 participants (5.6%) with other disorders (anorexia nervosa, somatization, and hypochondria disorders).

Eighty‐five (34.4%) of the sample were male and 162 (65.6%) of the total sample were female. The age range of the present sample was from 10 to 50 years with an average age of 29.8 years and a standard deviation of 8.62. Independent *t*‐test of age difference between the two groups showed that there was no significant difference between the groups in terms of age (*t* = 1.67; sig = .096). Marital status of the samples included 55.5% single, 36.4% married, and 8.1% divorced. Education was 30.8% diploma, 19.4% associate, 23.5% bachelor's degree, and 26.3% master's degree and above. Thirty six percent were unemployed, 34% were full‐time, 29.6% were part‐time, and 0.4 were self‐employed. Fifty participants (20.2%) had a history of medical illness, and 90 participants (36.4%) had a history of psychiatric and psychological disorders, of which 4 patients (1.6%) had received medical treatment and 31 participants (12.6%) had received psychological treatment. Fifty‐five (22.3%) of these participants had received medical and psychological treatment, and as the results of Table 1 show, there was no significant difference between the anxiety disorders and non‐clinical groups in the variables of gender, education, occupation, and history of medical disorder. The history of psychiatric and psychological disorders was significant between the two groups, which was normal due to the nature of the groups, and the marital status of the two groups was also significant.

- **Formal and content validity of the SCAARED**:

**TABLE 1:** Frequency and percentage for demographic characteristics

		Non‐clinical		Anxiety disorders		Chi‐square	
Variable	Groups	Frequency	Percentage	Frequency	Percentage	Value	Sig
Gender	Male	45	52.9	40	47.1	0.738	.39
	Female	95	58.6	67	47.4		
marital status	Single	74	54	63	46	16.819	.000
	Married	62	68.9	28	31.1		
	Divorced	4	20	16	80		
Education status	Diploma	39	51.3	37	48.7	3.317	.345
	Associate	31	64.6	17	35.4		
	Bachelor	30	51.7	28	48.3		
	Masters and higher	40	61.5	25	38.5		
Employment status	Unemployed	49	55.1	40	44.9	0.263	.877
	Freelance job	41	56.2	32	43.8		
	Employee	50	58.8	35	41.2		
History of serious physical illness		24	48	26	52	1.924	.165
History of psychiatric disorders		140	56.7	107	43.3	171.363	.000
History of treatment	Pharmacological	0	0	4	4.4	3.893	.143
	Psychotherapy	2	2.2	29	32.2		
	Pharmacological and psychotherapy	0	0	55	61.1		

To quantify face validity, the effect size of each question was calculated. For this purpose, the translated original version was provided to five psychologists and psychiatrists to evaluate items such as compatibility of the translated text with the original text, comprehensibility for the subjects, and the order of the question. The quantitative effect method of the item was used to reduce and eliminate inappropriate phrases and determine the importance of each phrase. For this purpose, for each of the 44 tool items, a Likert scale from 1 to 4 was considered, which showed a higher importance of the item. After completing the questionnaire by experts, face validity was calculated using the formula of item effect method. The impact score of the questions was between 4 and 5, so the face validity of all the questions was accepted by the evaluators.

In order to check the content validity, the opinions of the experts that belonged to the essential option were quantified through the CVR. The questions were accepted or rejected based on the CVR; if the CVR of the question was equal to or greater than 0.75, the question was accepted unconditionally. The CVI is obtained by dividing the number of experts who have given a score of 3 or 4 to the total number of experts. CVI acceptance score above 0.79 is considered appropriate. After collecting the questionnaires from the experts and entering the information into the software, CVR values were calculated for each question and also for the whole questionnaire, which was equal to 0.86. The final CVI value was 0.85, which was 0.83 for simplicity, 0.92 for relevance, and 0.80 for clarity.

- **Construct validity of the SCAARED**:

In studies that construct or evaluate the validity and reliability of tools, in order to examine the compatibility of the results obtained from the metrics with the theories on which the test is designed, it is necessary to examine the validity of the structure. Examining the factor structure of the test through factor analysis methods is the most well‐known method of construct validity. Factor analysis is done in two ways: exploratory and confirmatory. Exploratory factor analysis is used when the researcher does not have sufficient prior and pre‐empirical evidence to form a hypothesis about the number of factors underlying the data and is actually willing to dig into the data to determine the number or nature of factors that justify the overlap between variables. In confirmatory factor analysis, the researcher's goal is to confirm a specific factor structure, hypotheses about the number of factors are clearly stated, and the fit of the desired factor structure in the hypothesis with the covariance structure of the measured variables is tested (Sarmad et al., 2004).

Therefore, at first, the present study investigated the construct validity of the SCAARED through confirmatory factor analysis by LISREL software. Given that there is no general agreement among structural equation modeling experts as to which of the fit indices provides a better estimate of the model, it is suggested that a combination of three to four indices be reported. As a result, in the present study, in line with the main validation studies of the SCAARED, among the indicators of absolute fitness, the ratio of chi‐square to degree of freedom (x2/df), the index of good fit (GFI), and the root mean square of the approximate estimation square (RMSEA) and among the indices of comparative or comparative fitness, Tucker–Lewis fit index or non‐normed fit indexes fit index (NNFI) as well as adaptive fitness index (CFI) were used. The main manufacturers listed four factors for the SCAARED, which are determined by 44 items. The results obtained from confirmatory factor analysis by LISREL software showed that the fitness indicators of the 4‐factor model indicated the lack of proper validity of the model. Table 2 shows the fitting information of the proposed models, and considering that the software proposals to modify the results and achieve the optimal model were very different from the original model, the exploratory factor analysis method was used through principal component analysis with the inclined rotation method to achieve new factors.

**TABLE 2:** Fitting data of 4‐factor models of the SCAARED by confirmatory factor analysis method

Variable	χ 2	df	*p*	χ2/df	GFI	CFI	NNFI	RMSEA	SRMR
SCAARED	371.16	896	.000	4.16	0.59	0.94	0.93	0.113	0.081

The results of exploratory factor analysis showed that there are four factors with an eigenvalue higher than 0.3, which explains 66.19% of the total variance. Sample Kaiser–Meyer–Olkin (KMO) (0.925) and Bartlett test of sphericity (chi‐square equal to 10462.48 and degree of freedom 946 with significance of .001) showed that the sample and correlation matrix are suitable for this analysis. Table 3 shows the factor loads of each item in the corresponding factor.

- **Internal reliability, test–retest reliability, and convergent and divergent validity of the SCAARED**:

**TABLE 3:** Factor loads of Model 4 Factor of the SCAARED by exploratory factor analysis method

Factor					Factor				
4	3	2	1	Item	4	3	2	1	Item
		0.690		25				0.814	7
		0.683		32				0.805	29
		0.672		38				0.792	31
		0.665		28				0.789	23
		0.653		19				0.782	21
		0.547		18				0.778	24
		0.520		36				0.751	8
		0.491		9				0.742	44
	0.835			26				0.742	5
	0.834			20				0.731	39
	0.811			13				0.719	14
	0.791			4				0.707	37
	0.770			16				0.667	35
	0.658			33				0.501	22
	0.651			30			0.833		2
0.855				27			0.790		17
0.780				10			0.781		40
0.768				34			0.774		1
0.763				43			0.763		12
0.756				42			0.710		6
0.744				41			0.704		15
0.702				3			0.704		11

Examination of the standardized Cronbach's alpha of items to measure the internal reliability of the questionnaire showed that the SCAARED has a reliability of 0.966 and therefore the Persian version of this questionnaire is a reliable tool. The results of the correlation (Table 4) show that there was a strong correlation at the level (*p* < .01) between the questionnaire factors and the factors with the total score, and a strong correlation between the first and second performance in the four factors and the overall score. Therefore, it can be concluded that the test has good test–retest reliability. Also, as the results in Table 5 show, there is a positive correlation (*p *< .01) between the factors and the overall score of the SCAARED with anxiety disorders measured based on SCID‐5‐CV (*p* < .01), which indicates optimal convergent validity. There is a negative correlation between the factors and the overall score of the SCAARED with the PWI‐A at the level (*p* < .01), which indicates the favorable divergent validity of the SCAARED.

**TABLE 4:** Correlation between factors and first and second implementation

	GA	PA/SO	SOC	SEP	TOTAL	RE.GA	RE.PA/SO	RE.SOC	RE.SEP
GA	1								
PA/SO	**0.638	1							
SOC	**0.422	**0.423	1						
SEP	**0.358	**0.548	**0.450	1					
TOTAL	**0.848	**0.885	**0.638	**0.702	1				
RE.GA	**0.896	**0.556	**0.565	*0.230	**0.760	1			
RE.PA/SO	**0.645	**0.918	**0.507	**0.678	**0.897	**0.598	1		
RE.SOC	**0.597	**0.514	**0.851	**0.257	**0.676	**0.548	**0.467	1	
RE.SEP	**0.253	**0.659	**0.292	**0.917	**0.643	*0.215	**0.684	**0.288	1
RE.Total	**0.810	**0.862	**0.674	**0.644	**0.966	**0.815	**0.910	**0.678	**0.667

* *p* < .05.

** *p* < .01.

**TABLE 5:** Correlation between factors of the SCAARED with PWI‐A and anxiety disorders

	GAD	SAD	Separation AD	Panic	PWI‐A
GA	0.786**	0.405**	0.200**	0.589**	0.529**
PA/So	0.660**	0.566**	0.237**	0.663**	0.568**
SOC	0.510**	0.365**	0.536**	0.413**	0.405**
SEP	0.410**	0.504**	0.267**	0.386**	0.633**
Total	0.787**	0.688**	0.372**	0.662**	0.677**

** *p* < .01.

## 4 DISCUSSIONS

In this study, the psychometric properties of SCAARED, a self‐report scale for the screening of anxiety disorders in the adult population, were evaluated. In summary, SCAARED is a new tool with good psychometric properties for use in research and clinical practice as well as a promising tool for screening anxiety disorders in a variety of treatment settings.

The results of the present study confirm the face and content validity of the SCAARED. A review of the Cronbach's standardized alpha of the questions to measure the internal reliability of the instrument showed that SCAARED has a good reliability and therefore the Persian version of these questionnaires is a reliable tool. Also, the results showed a strong correlation between the first and second implementation in four factors and the overall score. Therefore, it can be concluded that the SCAARED has good test–retest reliability. Also, there is a positive correlation between the factors and the overall score of the SCAARED scale with anxiety disorders based on SCID‐5‐CV, which indicates the favorable convergent validity of the SCAARED. There is a negative correlation between the factors and the overall score of the SCAARED with the PWI‐A at the level, which indicates the favorable divergent validity of the SCAARED, and the results of exploratory factor analysis of the SCAARED were confirmed.

The results of the present study are in line with the findings of the only study reviewed of the SCAARED. In this study, SCAARED had a good internal consistency and four factors were reported: somatic/panic/agoraphobia, generalized anxiety, social anxiety, and separation anxiety. In fact, these factors are consistent with the diagnostic classes in the DSM‐5. Also, the results of this study showed good discriminant validity between participants with and without anxiety disorder in two independent samples. Also, despite the usual comorbidity of anxiety and depression, SCAARED separated participants with anxiety and participants with depression only. Individual and general analysis scores were more significant in women. Individual and total SCAARED analysis scores showed stability over time (Angulo et al., 2017). These results suggest that SCAARED is a suitable tool for screening for anxiety disorders in the adult population. SCAARED is sensitive to response to treatment (Compton, Peris, et al. 2014; Compton, Walkup, et al., 2014) and biological changes in the brain (Perlman et al., 2014). In future studies, SCAARED could be useful for screening for anxiety disorders in psychiatric settings and primary care and psychiatric clinics, and In future studies, SCAARED could be useful for screening for anxiety disorders in psychiatric settings and primary care and psychiatric clinics.

One of the limitations of the present study is that SCAARED was performed on a sample of the Iranian population in a specific geographical area. Therefore, it is suggested that this tool be examined in other communities and with a wider sample to generalize the results more accurately. Also, in some diagnostic stages, anxiety disorder was a small sample, and therefore, it is suggested to compare the results in another group considering the higher sample size in anxiety disorder groups. SCAARED does not include specific phobias and all people with agoraphobia in this questionnaire with panic disorder are on the same factor. However, given that agoraphobia without panic disorder is less common than agoraphobia without panic disorder (Kessler et al., 2005), SCAARED is a useful tool for screening patients with any of these disorders. For patients with high scores on the panic/agoraphobia factor, it is recommended that specialists consider items related to agoraphobia (Bramley et al., 1988; Reynolds et al., 2003; Zimmerman & Mattia, 1999).

## 5 CONCLUSIONS

Based on the findings of this study, SCAARED is a tool with appropriate validity and reliability. This tool can be used in psychiatric and psychological clinics for screening patients with anxiety disorders in accordance with the diagnoses presented in DSM‐5, as well as in research, clinical, and therapeutic practice to assess the patients during treatment, the outcome of treatment, and the improvement of patients' symptoms.

## CONFLICT OF INTEREST

The authors declare that there is no conflict of interest that could be perceived as prejudicing the impartiality of the research reported.

### PEER REVIEW

The peer review history for this article is available at https://publons.com/publon/10.1002/brb3.2647

## Data Availability

The Persian version of the Screen for adult anxiety related disorders (SCAARED) is a tool with appropriate validity and reliability. The data that support the findings of this study are available from the corresponding author upon reasonable request.
